# Supplementary material for: Anomalous circular bulk photovoltaic effect in BiFeO3 thin films with stripe-domain pattern
Source: Nat Commun. 2021 Jan 12;12:282. doi: 10.1038/s41467-020-20446-z (PMC7804139; doi:10.1038/s41467-020-20446-z)
Supplement: Supplementary file 1 — Supplementary Information [file 41467_2020_20446_MOESM1_ESM.pdf]

Supplementary Material: Anomalous Circular Bulk Photovoltaic Effect in $BiFeO_3$ Thin Films with Stripe-Domain Pattern

David S. Knoche[1,2], Matthias Steimecke[3], Yeseul Yun[1,2], Lutz Mühlenbein[1,2], and Akash Bhatnagar[1,2*]

[1] *Zentrum für Innovationskompetenz SiLi-nano, Martin-Luther-Universität Halle-Wittenberg, Halle (Saale), 06120, Germany*
[2] *Institut für Physik, Martin-Luther-Universität Halle-Wittenberg, Halle (Saale), 06120, Germany*
[3] *Institut für Chemie, Technische Chemie, Martin-Luther-Universität Halle-Wittenberg, Halle (Saale), 06120, Germany*

[*] *akash.bhatnagar@physik.uni-halle.de*

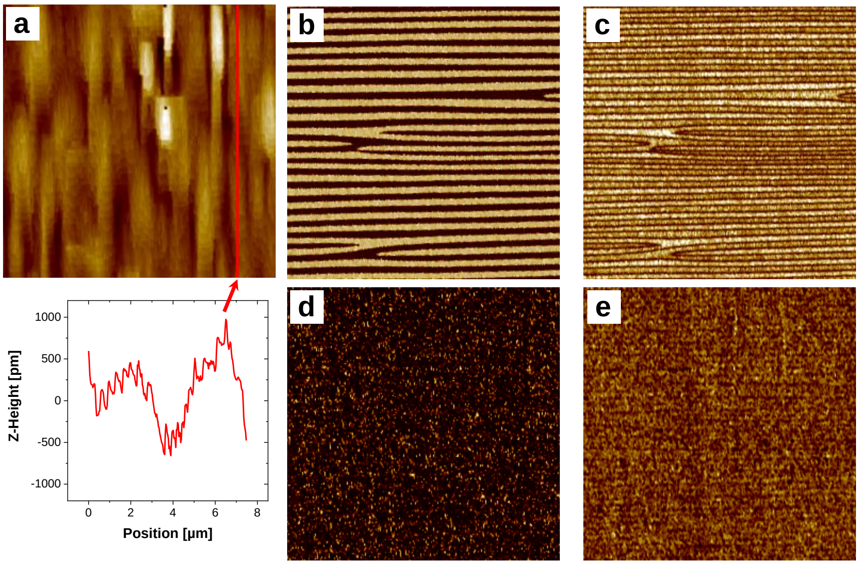

Figure 1: Scanning Probe Microscopy. a Atomic force microscopy image revealing the surface of a $BiFeO_3/DyScO_3$ thin film (top) and line scan of marked line (bottom). Lateral **b** phase and **c** amplitude piezo-response force microscopy (PFM) images. Vertical **d** phase and **e** amplitude PFM image. All images acquired simultaneously with a scanning area of $7.5 \times 7.5\,\mu m^2$.

1 Tensorial Analysis of the BPV Effect in $BiFeO_3$

1.1 Derivation of BPV tensors

Application of symmetry operation for point group 3m to general 3rd rank tensor

Symmetry operations matrices:

Threefold rotation (3) parallel to Z_3 and mirror (m) parallel to Z_1:

$$3 \parallel Z_3 \begin{pmatrix} -\frac{1}{2} & \frac{\sqrt{3}}{2} & 0 \\ -\frac{\sqrt{3}}{2} & -\frac{1}{2} & 0 \\ 0 & 0 & 1 \end{pmatrix},\ m \perp Z_1 \begin{pmatrix} -1 & 0 & 0 \\ 0 & 1 & 0 \\ 0 & 0 & 1 \end{pmatrix} \tag{1}$$

Resulting 3rd rank tensor: [1]

$$\left(\begin{array}{ccc|ccc|ccc} 0 & \beta_{222} & \beta_{223} & \text{-}\beta_{222} & 0 & 0 & \beta_{311} & 0 & 0 \\ \text{-}\beta_{222} & 0 & 0 & 0 & \beta_{222} & \beta_{223} & 0 & \beta_{311} & 0 \\ \beta_{131} & 0 & 0 & 0 & \beta_{131} & 0 & 0 & 0 & \beta_{333} \end{array}\right) \tag{2}$$

Phenomenological expression of BPV [2]

The BPV effect can be described with following equation:

$$j_{\mathrm{i}} = I\beta_{\mathrm{ijk}} E_{\mathrm{j}} E^{*}_{\mathrm{k}} \tag{3}$$

The real part of β_{ijk} is symmetric and imaginary part of β_{ijk} is antisymmetric:

$$\mathrm{Re}\,\beta_{\mathrm{ijk}} = \mathrm{Re}\,\beta_{\mathrm{ikj}} = \beta^{\mathrm{L}}_{\mathrm{ijk}};\ \ \mathrm{Im}\,\beta_{\mathrm{ijk}} = -\mathrm{Im}\,\beta_{\mathrm{ikj}} = \beta^{\mathrm{C}}_{\mathrm{ijk}} \tag{4}$$

The current expression becomes:

$$j_{\mathrm{i}} = j^{\mathrm{L}}_{\mathrm{i}} + j^{\mathrm{C}}_{\mathrm{i}} = I\left(\beta^{\mathrm{L}}_{\mathrm{ijk}} + i\beta^{\mathrm{C}}_{\mathrm{ijk}}\right) E_{\mathrm{j}} E^{*}_{\mathrm{k}} \tag{5}$$

The nonsymmetric elements of the general 3rd rank tensor can be rewritten as having symmetric and antisymmetric parts. This lead to the symmetric part (LBPV) and antisymmetric part (CBPV).

Separation of 3rd rank tensor in symmetric and antisymmetric part

$$\beta^{\mathrm{L}}_{\mathrm{ijk}} = \left(\begin{array}{ccc|ccc|ccc} 0 & \text{-}\beta^{\mathrm{L}}_{222} & \beta^{\mathrm{L}}_{131} & \text{-}\beta^{\mathrm{L}}_{222} & 0 & 0 & \beta^{\mathrm{L}}_{311} & 0 & 0 \\ \text{-}\beta^{\mathrm{L}}_{222} & 0 & 0 & 0 & \beta^{\mathrm{L}}_{222} & \beta^{\mathrm{L}}_{131} & 0 & \beta^{\mathrm{L}}_{311} & 0 \\ \beta^{\mathrm{L}}_{131} & 0 & 0 & 0 & \beta^{\mathrm{L}}_{131} & 0 & 0 & 0 & \beta^{\mathrm{L}}_{333} \end{array}\right) \tag{6}$$

$$\beta^{\mathrm{C}}_{\mathrm{ijk}} = \left(\begin{array}{ccc|ccc|ccc} 0 & 0 & \text{-}\beta^{\mathrm{C}}_{131} & 0 & 0 & 0 & 0 & 0 & 0 \\ 0 & 0 & 0 & 0 & 0 & \text{-}\beta^{\mathrm{C}}_{131} & 0 & 0 & 0 \\ \beta^{\mathrm{C}}_{131} & 0 & 0 & 0 & \beta^{\mathrm{C}}_{131} & 0 & 0 & 0 & 0 \end{array}\right) \tag{7}$$

Transformation of LBPV (symmetric BPV) tensor into matrix notation [3, 4]

(6) is transformed is into the corresponding 3x6 matrix $\beta^{\mathrm{L}}_{\mathrm{il}}$.. If $l = 1, 2, 3$, then $\beta^{\mathrm{L}}_{\mathrm{il}} = \beta^{\mathrm{L}}_{\mathrm{ill}}$ and if $l = 4, 5, 6$, then $\beta^{\mathrm{L}}_{\mathrm{il}} = 2\beta^{\mathrm{L}}_{\mathrm{ijk}}$.

$$\beta^{\mathrm{L}}_{\mathrm{il}} = \begin{pmatrix} 0 & 0 & 0 & 0 & \beta^{\mathrm{L}}_{15} & -2\beta^{\mathrm{L}}_{22} \\ -\beta^{\mathrm{L}}_{22} & \beta^{\mathrm{L}}_{22} & 0 & \beta^{\mathrm{L}}_{15} & 0 & 0 \\ \beta^{\mathrm{L}}_{31} & \beta^{\mathrm{L}}_{31} & \beta^{\mathrm{L}}_{33} & 0 & 0 & 0 \end{pmatrix} \tag{8}$$

The appearance of $\beta^{\mathrm{L}}_{\mathrm{il}}$ is analogous to the piezoelectric tensor d_{ij}.

Transformation of CBPV (antisymmetric BPV) into 2nd rank pseudo-tensor [5]

(7) can be transformed into a pseudo-tensor using the Levi-Civita symbol ϵ_{ijk}:

$$\beta^{\mathrm{C}}_{\mathrm{li}} = \epsilon_{\mathrm{ijk}}\beta^{\mathrm{A}}_{\mathrm{ljk}} \tag{9}$$

The resulting 2nd rank pseudo-tensor is:

$$\beta^{\mathrm{C}}_{\mathrm{ij}} = \begin{pmatrix} 0 & +\beta^{\mathrm{C}}_{12} & 0 \\ -\beta^{\mathrm{C}}_{12} & 0 & 0 \\ 0 & 0 & 0 \end{pmatrix} \tag{10}$$

The appearance $\beta^{\mathrm{C}}_{\mathrm{ij}}$ is analogous to the gyrotropic axial (pseudo-)tensor g_{lk}, which is connected to the natural optical activity tensor $\gamma^{\mathrm{A}}_{\mathrm{ijl}}$ using the Levi-Civita symbol ϵ_{ijk}. [6, 7]

1.2 Tensorial calculation for ordered striped 71° domain pattern

Relation between coordinate system based on hexagonal and pseudo-cubic crystal system

The standard axes of physical properties follow the IEEE standard setting. For trigonal crystals, the convention is based on the hexagonal crystal system. Therefore, the z axis of physical properties is set along the c crystallographic axis. The x axis is set along the a crystallographic axis and the y axis is orthogonal to the other completing the right-hand cartesian coordinate system. To calculate the BPV response for the experimental setup, it is necessary to move to a coordinate system which is related to the used axis in the setup.

A three-dimensional coordinate system can be transformed into another arbitrary three-dimensional coordinate system using a 3x3 matrix. Regarding the symmetry relations shown in Figure 2, the coordinate system of the physical properties based on the hexagonal crystal system ($\mathrm{x_h}$, $\mathrm{y_h}$, $\mathrm{z_h}$) can be transformed into transformed into the pseudo-cubic coordinate system ($\mathrm{x_{pc}}$, $\mathrm{y_{pc}}$, $\mathrm{z_{pc}}$) using following matrix:

$$a_{\mathrm{ij}} = a_{\mathrm{P_L}} = \begin{pmatrix} 0 & \frac{2}{\sqrt{6}} & \frac{1}{\sqrt{3}} \\ -\frac{1}{\sqrt{2}} & -\frac{1}{\sqrt{6}} & \frac{1}{\sqrt{3}} \\ \frac{1}{\sqrt{2}} & -\frac{1}{\sqrt{6}} & \frac{1}{\sqrt{3}} \end{pmatrix} \tag{11}$$

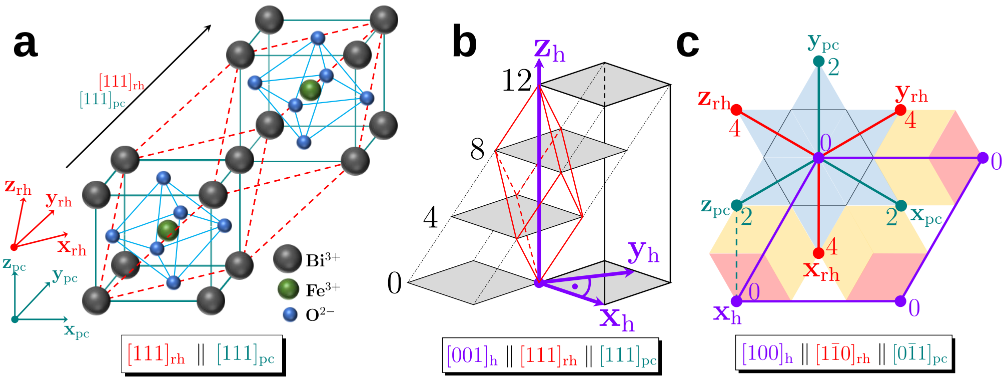

Figure 2: Coordinate system transformation between different crystal system. **a** Relation between pseudo-cubic and rhombohedral coordinate system. [8] The rhombhohedral unit cell can be described with two ABO_3 cubic unit cells. **b** Relation between rhombhohedral and hexagonal crystal system (Adapted from [9]). **c** Projection of (Adapted from [10]). The numbers depict the position height with respect to the hexagonal unit cell (unit: $c_h/12$).

The index $\mathrm{P_L}$ refers to the domain variants $\mathrm{P_L}$ (Figure 1 (c),(d) main text)) with the polarization pointing along the $[111]_{\mathrm{pc}}$ direction. To describe the response for the domain variants $\mathrm{P_R}$ the resulting response has be rotated by +90° (CCW) around the $[001]_{\mathrm{pc}}$ direction:

$$a_{\mathrm{P_R}} = \begin{pmatrix} \cos(\frac{\pi}{2}) & \sin(\frac{\pi}{2}) & 0 \\ -\sin(\frac{\pi}{2}) & \cos(\frac{\pi}{2}) & 0 \\ 0 & 0 & 1 \end{pmatrix} a_{\mathrm{P_L}} = \begin{pmatrix} 0 & 1 & 0 \\ -1 & 0 & 0 \\ 0 & 0 & 1 \end{pmatrix} a_{\mathrm{P_L}} = \begin{pmatrix} -\frac{1}{\sqrt{2}} & -\frac{1}{\sqrt{6}} & \frac{1}{\sqrt{3}} \\ 0 & -\frac{2}{\sqrt{6}} & -\frac{1}{\sqrt{3}} \\ \frac{1}{\sqrt{2}} & -\frac{1}{\sqrt{6}} & \frac{1}{\sqrt{3}} \end{pmatrix} \quad (12)$$

LBPV response

For light wave with linear polarization propagating along $[001]_{\mathrm{pc}}$:

$$(E_{\mathrm{j}})^{\mathrm{pc}}_{\mathrm{L}} = \begin{pmatrix} \cos\theta \\ \sin\theta \\ 0 \end{pmatrix}, (E_{\mathrm{j}})^{\mathrm{h}}_{\mathrm{L}} = a^{\mathrm{T}}_{\mathrm{L}} (E_{\mathrm{j}})^{\mathrm{pc}}_{\mathrm{L}} = \begin{pmatrix} -\frac{\sin(\theta)}{\sqrt{2}} \\ \frac{2\cos(\theta)}{\sqrt{6}} - \frac{\sin(\theta)}{\sqrt{6}} \\ \frac{\sin(\theta)}{\sqrt{3}} + \frac{\cos(\theta)}{\sqrt{3}} \end{pmatrix} \quad (13)$$

The orientation of the light wave with respect to the R-configuration is shifted by 90° clockwise, which leads to:

$$(E_{\mathrm{j}})^{\mathrm{pc}}_{\mathrm{R}} = \begin{pmatrix} \cos(\theta + \frac{\pi}{2}) \\ \sin(\theta + \frac{\pi}{2}) \\ 0 \end{pmatrix} = E^{\mathrm{pc}}_{\mathrm{j}} = \begin{pmatrix} -\sin\theta \\ \cos\theta \\ 0 \end{pmatrix}, (E_{\mathrm{j}})^{\mathrm{h}}_{\mathrm{R}} = a^{\mathrm{T}}_{\mathrm{L}} (E_{\mathrm{j}})^{\mathrm{pc}}_{\mathrm{R}} = \begin{pmatrix} -\frac{\cos(\theta)}{\sqrt{2}} \\ -\frac{2\sin(\theta)}{\sqrt{6}} - \frac{\cos(\theta)}{\sqrt{6}} \\ \frac{\cos(\theta)}{\sqrt{3}} - \frac{\sin(\theta)}{\sqrt{3}} \end{pmatrix} \quad (14)$$

The overall response in the pseudo-cubic coordinate system can be now calculated:

$$j_{\mathrm{i}}^{\mathrm{L}} = \frac{I}{2}\left[\left[a_{\mathrm{R}}[\beta_{\mathrm{ij}}^{\mathrm{L}}[(EE)_{\mathrm{j}}]_{\mathrm{R}}^{\mathrm{H}}] + a_{\mathrm{L}}[\beta_{\mathrm{ij}}^{\mathrm{L}}[(EE)_{\mathrm{j}}]_{\mathrm{L}}^{\mathrm{H}}]\right]\right]$$

$$= I \begin{pmatrix} \left(\frac{\beta_{33}^{\mathrm{L}}}{3\sqrt{3}} + \frac{2\beta_{31}^{\mathrm{L}}}{3\sqrt{3}} + \frac{\beta_{22}^{\mathrm{L}}}{3\sqrt{6}} + \frac{\beta_{15}^{\mathrm{L}}}{6\sqrt{3}}\right) + \left(\frac{\beta_{22}^{\mathrm{L}}}{\sqrt{6}} + \frac{\beta_{15}^{\mathrm{L}}}{2\sqrt{3}}\right)\cos(2\theta) \\ \left(\frac{\beta_{33}^{\mathrm{L}}}{3\sqrt{3}} - \frac{\beta_{31}^{\mathrm{L}}}{3\sqrt{3}} - \frac{2\beta_{22}^{\mathrm{L}}}{3\sqrt{6}} + \frac{\beta_{15}^{\mathrm{L}}}{6\sqrt{3}}\right)\sin(2\theta) \\ \left(\frac{2\beta_{22}^{\mathrm{L}}}{3\sqrt{6}} + \frac{\beta_{15}^{\mathrm{L}}}{3\sqrt{3}} - \frac{\beta_{33}^{\mathrm{L}}}{3\sqrt{3}} - \frac{2\beta_{31}^{\mathrm{L}}}{3\sqrt{3}}\right) \end{pmatrix} \tag{15}$$

CBPV response

The circularly-polarized light propagates in $[001]_{\mathrm{pc}}$ direction.

Left-handed circularly polarized light (LCP)

Jones vector:

$$\mathbf{e}_{\mathrm{LCP,pc}} = \begin{pmatrix} \frac{1}{\sqrt{2}} \\ \frac{i}{\sqrt{2}} \\ 0 \end{pmatrix}, \mathbf{e}_{\mathrm{LCP,h}} = \begin{pmatrix} -\frac{i}{2} \\ \frac{\sqrt{2}}{\sqrt{6}} - \frac{i}{\sqrt{2}\sqrt{6}} \\ \frac{1}{\sqrt{2}\sqrt{3}} + \frac{i}{\sqrt{2}\sqrt{3}} \end{pmatrix} \tag{16}$$

CBPV response for LCP light for each domain variant:

$$j_{\mathrm{i,P_L}}^{\mathrm{LCP}} = \frac{I}{2} a_{\mathrm{P_L}} \beta_{\mathrm{ij}}^{\mathrm{C}} i\left[\mathbf{e}_{\mathrm{LCP,h}} \times \mathbf{e}_{\mathrm{LCP,h}}^{*}\right] = \frac{I}{2\sqrt{3}} \begin{pmatrix} -\beta_{12}^{\mathrm{C}} \\ +\beta_{12}^{\mathrm{C}} \\ 0 \end{pmatrix} \tag{17}$$

$$j_{\mathrm{i,P_R}}^{\mathrm{LCP}} = \frac{I}{2} a_{\mathrm{P_R}} \beta_{\mathrm{ij}}^{\mathrm{C}} i\left[\mathbf{e}_{\mathrm{LCP,h}} \times \mathbf{e}_{\mathrm{LCP,h}}^{*}\right] = \frac{I}{2\sqrt{3}} \begin{pmatrix} +\beta_{12}^{\mathrm{C}} \\ +\beta_{12}^{\mathrm{C}} \\ 0 \end{pmatrix} \tag{18}$$

Overall CBPV response for LCP light:

$$j_{\mathrm{i}}^{\mathrm{LCP}} = \frac{I}{2}\left[a_{\mathrm{P_R}}\left(\beta_{\mathrm{ij}}^{\mathrm{C}} i\left[\mathbf{e}_{\mathrm{LPC,h}} \times \mathbf{e}_{\mathrm{LCP,h}}^{*}\right]\right) + a_{\mathrm{P_L}}\left(\beta_{\mathrm{ij}}^{\mathrm{C}} i\left[\mathbf{e}_{\mathrm{LPC,h}} \times \mathbf{e}_{\mathrm{LCP,h}}^{*}\right]\right)\right] = \frac{I}{\sqrt{3}} \begin{pmatrix} 0 \\ +\beta_{12}^{\mathrm{C}} \\ 0 \end{pmatrix} \tag{19}$$

Right-handed circularly polarized light (RCP)

$$\mathbf{e}_{\mathrm{RCP,pc}} = \begin{pmatrix} \frac{1}{\sqrt{2}} \\ -\frac{i}{\sqrt{2}} \\ 0 \end{pmatrix}, \mathbf{e}_{\mathrm{RCP,h}} = \begin{pmatrix} \frac{i}{2} \\ \frac{\sqrt{2}}{\sqrt{6}} + \frac{i}{\sqrt{2}\sqrt{6}} \\ \frac{1}{\sqrt{2}\sqrt{3}} - \frac{i}{\sqrt{2}\sqrt{3}} \end{pmatrix} \tag{20}$$

CBPV response for RCP light for each domain variant:

$$j_{\mathrm{i,P_L}}^{\mathrm{RCP}} = \frac{I}{2} a_{\mathrm{P_L}} \beta_{\mathrm{ij}}^{\mathrm{C}} i\left[\mathbf{e}_{\mathrm{RCP,h}} \times \mathbf{e}_{\mathrm{RCP,h}}^{*}\right] = \frac{I}{2\sqrt{3}} \begin{pmatrix} +\beta_{12}^{\mathrm{C}} \\ -\beta_{12}^{\mathrm{C}} \\ 0 \end{pmatrix} \tag{21}$$

$$j^{\mathrm{RCP}}_{\mathrm{i,P_R}} = \frac{I}{2} a_{\mathrm{P_R}} \beta^{\mathrm{C}}_{\mathrm{ij}} i \left[\mathbf{e}_{\mathrm{RCP,h}} \times \mathbf{e}^{*}_{\mathrm{RCP,h}}\right] = \frac{I}{2\sqrt{3}} \begin{pmatrix} -\beta^{\mathrm{C}}_{12} \\ -\beta^{\mathrm{C}}_{12} \\ 0 \end{pmatrix} \tag{22}$$

Overall CBPV response for RCP light:

$$j^{\mathrm{RCP}}_{\mathrm{i}} = \frac{I}{2} \left[a_{\mathrm{P_R}} \left(\beta^{\mathrm{C}}_{\mathrm{ij}} i \left[\mathbf{e}_{\mathrm{RCP,h}} \times \mathbf{e}^{*}_{\mathrm{RCP,h}}\right]\right) + a_{\mathrm{P_L}} \left(\beta^{\mathrm{C}}_{\mathrm{ij}} i \left[\mathbf{e}_{\mathrm{RPC,h}} \times \mathbf{e}^{*}_{\mathrm{RCP,h}}\right]\right)\right] = \frac{I}{\sqrt{3}} \begin{pmatrix} 0 \\ -\beta^{\mathrm{C}}_{12} \\ 0 \end{pmatrix} \tag{23}$$

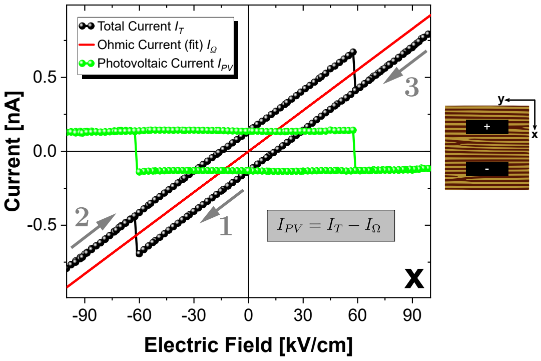

Figure 3: Switchability of the photovoltaic effect. Current as a function of applied electric field under illumination with linearly polarized laser light $\lambda = 405\,\mathrm{nm}$ in $[100]_{\mathrm{pc}}$ (black). At $\pm 60\mathrm{kV/cm}$, there is a abrupt current rise/drop. This apparent change is connected to the ferroelectric switching and therefore an opposing of the BPV orientation. By subtracting the ohmic conduction (red) from the overall response, a hysteretic behavior of the photovoltaic current (green) can be substracted.

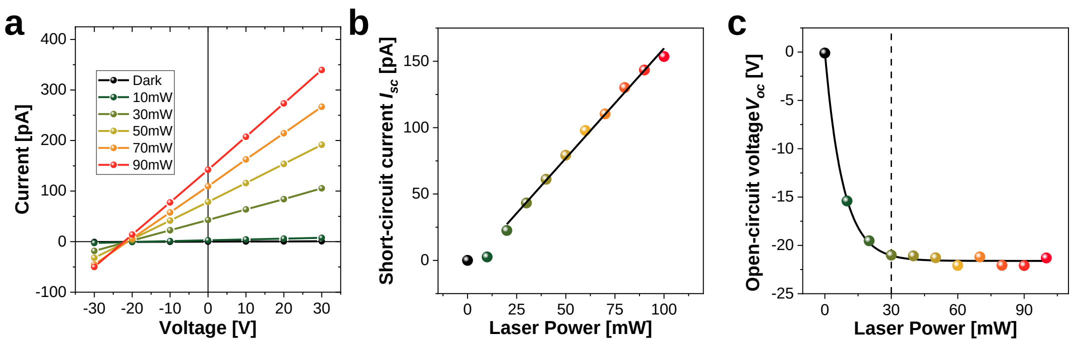

Figure 4: Photovoltaic properties as a function of light intensity. **a** Current-voltage characteristic under illumination with right-handed circularly polarized (LCP) light with different laser power settings. **b** Extracted short-circuit current I_{sc} and **c** open-circuit voltage V_{oc} as a function of the laser power.

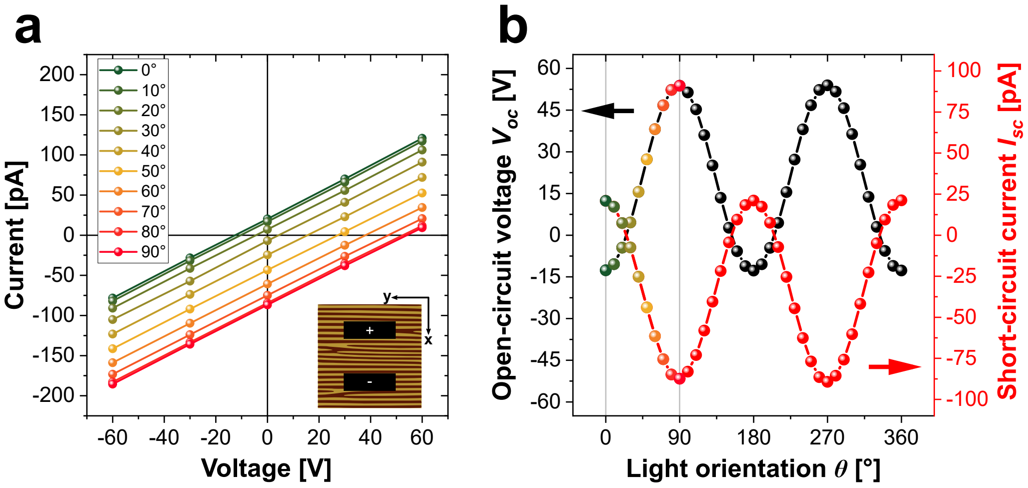

Figure 5: Extraction of photovoltaic properties from current-voltage characteristics. **a** Current-voltage (IV) characteristics for different orientation (0°‖**x**, CCW rotation) of the linearly polarized light. The electrode geometry is shown in the inset. **b** Open-circuit voltage V_{oc} and short-circuit current I_{sc} as a function of the light orientation θ. Both photovoltaic properties can be extracted from a linear fit of the IV characteristics.

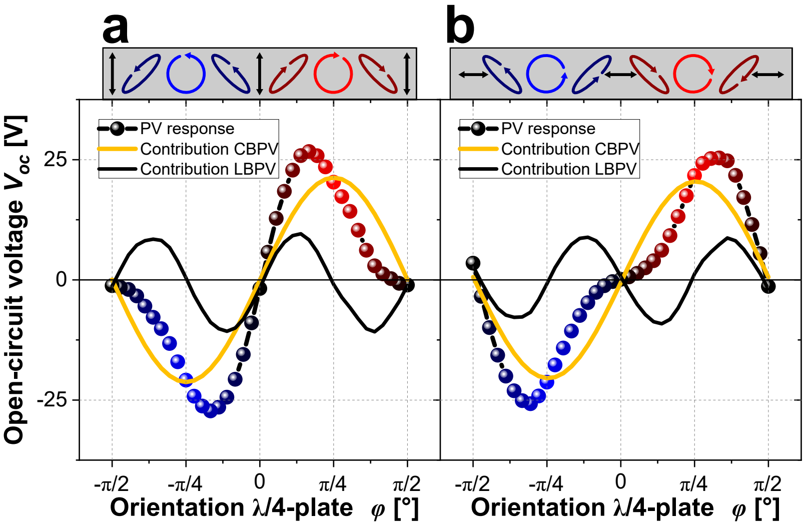

Figure 6: Comparison between different orientation of outcoming light polarization. Open-circuit voltage V_{oc} as a function of the $\lambda/4$ plate orientation ϕ for two different adjustment of the subsequent $\lambda/2$ plate. The starting linear light polarization aligned along **a** $[100]_{\mathrm{pc}}$ and **b** $[010]_{\mathrm{pc}}$, respectively (Compare schematics of the polarization state shown at the top).

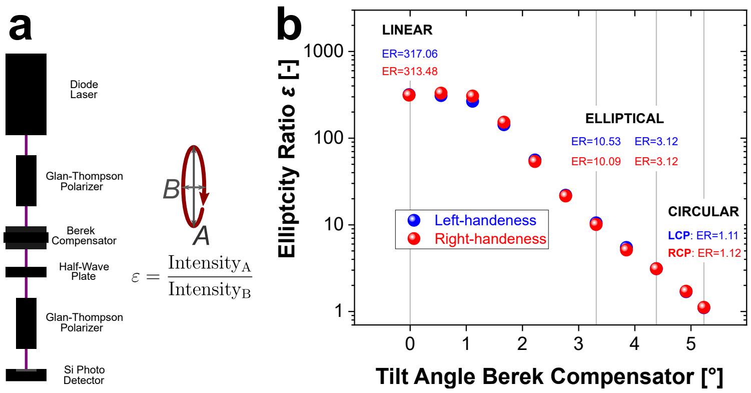

Figure 7: Light Polarization Characterization. **a** Schematic of the light polarization characterization setup. The set light polarization is rotated using the subsequent half-wave plate and the laser power for the major (A) and minor (B) axis is measured using a silicon photo detector (Thorlabs, DET100A2). **b** Ellipticity ratio ε as a function of the tilt angle of the Berek Compensator. Depending on the tilt angle, the pathway of the light through the birefringent MgF_2 plate is changed. This changes the resulting retardance and thus the outcoming light polarization from linear over elliptical to circular.

---

2 Raman Scattering Experiments

To explain the unexpected CBPV response in our films, we propose the existence of opposite circular dichroism for the different polarization variants. Consequently, CD spectra for each polarization variant is needed. To the best of our knowledge, commercially available CD spectrometer (mostly equipped with a Xe lamp) are not capable of resolving structure in the submicrometer range. However, circular dichroism has been observed using laser-based threshold photoemission electron microscopy (PEEM) in a $BiFeO_3$ single crystal with multi-domain state.[11]

Having this observation in mind, we conceived a Raman scattering experiment following partially the basic principle of Raman Optical Activity (ROA) in collaboration with our colleagues. The Raman spectra were acquired using a confocal Raman microscope setup (Renishaw, InVia). The sample was place on a **xy**-stage providing a 100 nm positioning resolution. The polarization state of the excitation laser light (532 nm) is tuned from linear to circular by inserting a quarter-wave plate. In order to gain information from a very localized region of the sample, we focused the laser beam to a spot size of ~1 µm. A spatial resolution below the laser spot size was achieved by using the StreamLine^{TM} high-resolution mode of the Raman instrument. In this mode, an increased spatial resolution is achieved by reducing the read-out area of the CCD detector during signal recording. A comparison between two spectra observed under illumination with linear (polarization along **x**-direction) and circular light polarization can be found in Figure 8(a). In both spectra, three dominant modes at 138 cm^{-1}, 174 cm^{-1} and 222 cm^{-1} were observed. This is in good agreement with previous studies on $BiFeO_3/DyScO_3$ thin films.[12] Contrarily, Talkenberger *et al.* used a 442 nm linearly polarized excitation

laser. Because of the smaller penetration depth of the light (~75 nm), they did not separate modes at higher wave numbers due to the $DyScO_3$ substrate (compare spectra of $DyScO_3$ substrate at bottom in Figure 8(a)). Because of this contribution from the substrate, we were not able to observe $BiFeO_3$ modes at higher wave numbers. In another study, Himcinshi *et al.* used Raman spectroscopy to identify domains with different polarization variants in $BiFeO_3$ a single crystal.[13] The change in the Raman signal (peak area of 172 cm^{-1} mode) due to a different orientation of the linear polarization of the excitation laser towards the corresponding polarization direction was used to map a domain configuration with micrometer-resolution. When performing the Raman scattering experiments, we observed a change of the ratio between the peak intensity of the 138 cm^{-1} and 174 cm^{-1} mode, however, under excitation with circularly polarized light. The intensity changes were not as high as shown by Himcinshi *et al.*[13], which might also be related to the smaller domain size and the different excitation wavelength.

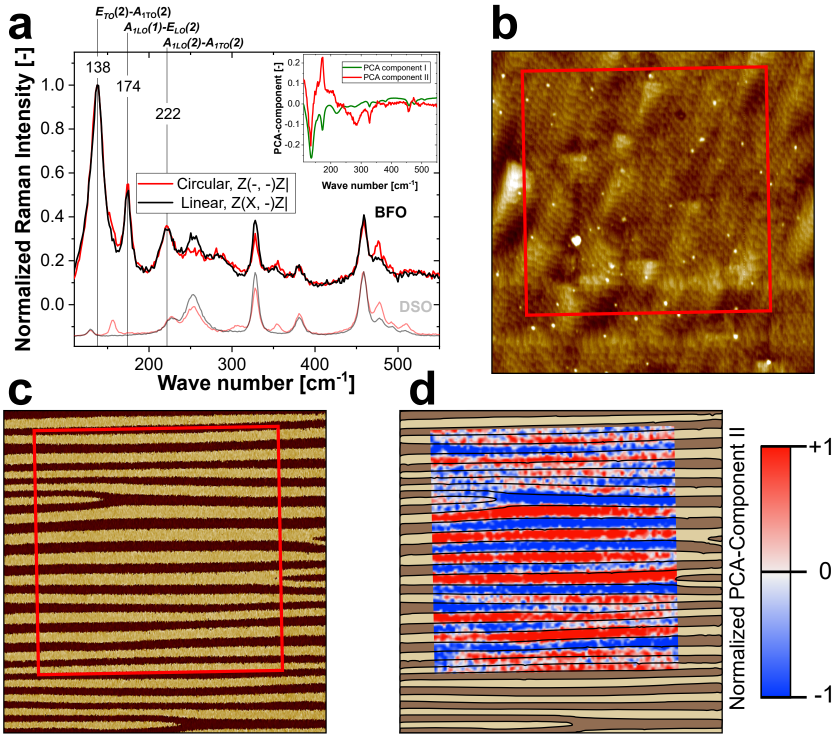

Figure 8: Raman scattering experiments demonstrating the domain-specific light-matter interaction. **a** Normalized Raman spectra of $BiFeO_3$/$DyScO_3$ thin film under circular (red) and linear (black) laser light excitation. Bottom: Qualitative Raman spectra of a $DyScO_3$ substrate. Inset: Both PCA-component used to analyze the spatial-resolved Raman spectra. **b** Topography and **c** in-plane PFM phase image of pre-characterized region (10×10 μm^2). The red squares indicates the position of the overlapped map acquired from the spatial-resolved Raman scattering experiments. **d** Normalized PCA-component II map (7.6×7.6 μm^2) acquired from the spatial-resolved Raman scattering experiments overlapping the schematic domain configuration extracted from PFM image in **c**. The outline of the map is inserted in **b** and **c** (red square).

To systematically investigate, this position-dependent change in the Raman spec-

tra, we characterized a 10×10 µm^2 region on our sample using piezo-response force microscopy (PFM). The AFM image (Figure 8(b)) reveals a topography with a similar roughness to the one shows in Figure 1(a) in our manuscript. The in-plane PFM (IP-PFM) phase image (Figure 8(c)) show great similarities to Figure 1(b), however, we want to mention the larger domain width in this region (∼250 nm compared to ∼150 nm in Figure 1(b) main text).

Then, we performed spatial-resolved Raman scattering experiments to acquire a two-dimensional grid of Raman spectra in a 8×8 µm^2 region in the vicinity of the PFM pre-characterized region. The Raman data (∼6400 spectra) were processed using principle component analysis (PCA)(WiRE 3.4 software, Renishaw), a commonly used tool show data variance. The PCA-analysis divides the Raman spectra into two main components (see inset Figure 8a). The change of the 174 cm^{-1} and 138 cm^{-1} peak intensity ratio can be represented by the PCA- component II. As a result, the position-dependent PCA-component can be mapped. To compare two characterization techniques, the resultant PCA-component map is overlapped with the IP-PFM phase image Figure 8(d). To match the shape of both pattern, the PCA-component map is resized (8×8 µm^2 → 7.6×7.6 µm^2). The lowest spatial resolution step of **xy**-positioning stage of the Raman microscope was used and a systemic positioning error while working at the lowest resolution limit could explain the necessity to resize the map to achieve a perfect overlap. However, the spatial resolution of this experiment is limited to $\sim\lambda/2$. For thinner structures, a mixed Raman spectra is obtained and it is not possible to clearly distinguish the different domains (compare Figure 8d upper and lower areas).

The possibility to resolve the ferroelectric domain arrangement using Raman scattering experiments under excitation with CP light suggests differential interaction between CP light and the domains exhibiting different polarization variants.

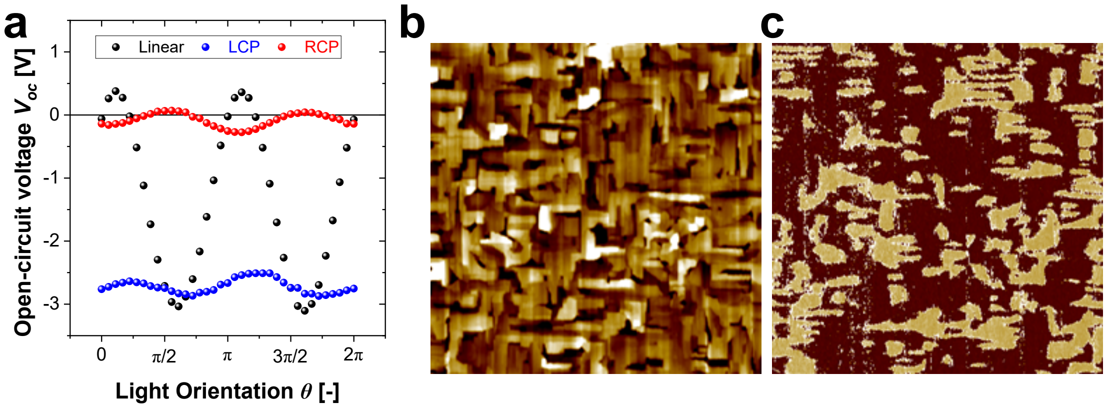

Figure 9: Linear and circular bulk photovoltaic effect in $BiFeO_3/SrTiO_3$ thin film. **a** Open-circuit voltage as a function of the light orientation θ for $BiFeO_3/SrTiO_3$ under illumination with linearly polarized (LP) light (black), left-handed circularly (LCP) light (blue) and right-handed-circularly polarized (RCP) light (red). **b** Atomic Force Microscopy image and **c** lateral piezo-response Force Microscopy phase image of $BiFeO_3/SrTiO_3$ sample. All images $7.5 \times 7.5\,\mu m^2$.

References

[1] D. W. Wilson, E. N. Glytsis, N. F. Hartman, and T. K. Gaylord, "Beam diameter threshold for polarization conversion photoinduced by spatially oscillating bulk photovoltaic currents in $LiNbO_3$:Fe," *Journal of the Optical Society of America B*, vol. 9, no. 9, p. 1714, 1992.

[2] V. I. Belinicher and B. I. Sturman, "The photogalvanic effect in media lacking a center of symmetry," *Soviet Physics Uspekhi*, vol. 23, no. 3, pp. 199–223, 1980.

[3] R. E. Newnham, *Properties of materials: Anisotropy, symmetry, structure.* Oxford: Oxford Univ. Press, 2004.

[4] J. F. Nye, *Physical properties of crystals: Their representation by tensors and matrices.* Oxford science publications, Oxford: Clarendon Press, reprinted. ed., 2012.

[5] B. I. Sturman and V. M. Fridkin, *The photovoltaic and photorefractive effects in noncentrosymmetric materials.* Philadelphia: Gordon and Breach, 1992.

[6] S. V. Gallego, J. Etxebarria, L. Elcoro, E. S. Tasci, and J. M. Perez-Mato, "Automatic calculation of symmetry-adapted tensors in magnetic and non-magnetic materials: a new tool of the bilbao crystallographic server," *Acta crystallographica. Section A, Foundations and advances*, vol. 75, no. Pt 3, pp. 438–447, 2019.

[7] L. D. Landau and E. M. Lifshits, *Electrodynamics of continuous media*, vol. 8 of *Course of theoretical physics.* Oxford: Pergamon, 2nd ed., rev ed., 1984.

[8] F. Kubel and H. Schmid, "Structure of a ferroelectric and ferroelastic monodomain crystal of the perovskite $BiFeO_3$," *Acta Crystallographica Section B Structural Science*, no. 46(6), pp. 698–702, 1990.

[9] M. J. Buerger, *X-ray crystallography: An introduction to the investigation of crystals by their diffraction of monochromatic x-radiation.* New York: Wiley, 3. print ed., 1953.

[10] H. D. Megaw and C. N. W. Darlington, "Geometrical and structural relations in the rhombohedral perovskites," *Acta Crystallographica Section A*, vol. 31, no. 2, pp. 161–173, 1975.

[11] A. Sander, M. Christl, C.-T. Chiang, M. Alexe, and W. Widdra, "Domain imaging on multiferroic bifeo 3 (001) by linear and circular dichroism in threshold photoemission," *Journal of Applied Physics*, vol. 118, no. 22, p. 224102, 2015.

[12] A. Talkenberger, I. Vrejoiu, F. Johann, C. Röder, G. Irmer, D. Rafaja, G. Schreiber, J. Kortus, and C. Himcinschi, "Raman spectroscopic investigations of epitaxial bifeo 3 thin films on rare earth scandate substrates," *Journal of Raman Spectroscopy*, vol. 46, no. 12, pp. 1245–1254, 2015.

[13] C. Himcinschi, J. Rix, C. Röder, M. Rudolph, M.-M. Yang, D. Rafaja, J. Kortus, and M. Alexe, "Ferroelastic domain identification in bifeo3 crystals using raman spectroscopy," *Scientific reports*, vol. 9, no. 1, p. 379, 2019.
